# Acute Deep Hand Burns Covered by a Pocket Flap-Graft: Long-term Outcome Based on Nine Cases

**Published:** 2007-01-16

**Authors:** Jean-Philippe Pradier, Christophe Oberlin, Eric Bey

**Affiliations:** a Plastic and Maxillo-Facial Surgery Service, Centre de Traitement des Brûlés, Hôpital d'instruction des Armées Percy, Clamart, France; b Paris Anatomy Institute, Institut d'Anatomie de Paris, Paris, France

## Abstract

**Objective:** We evaluated the long-term outcome of the “pocket flap-graft” technique, used to cover acute deep burns of the dorsum of the hand, and analyzed surgical alternatives. **Methods:** This was a 6-year, retrospective study of 8 patients with extensive burns and 1 patient with a single burn (11 hands in all) treated by defatted abdominal wall pockets. We studied the medical records of the patients, and conducted a follow-up examination. **Results:** All hands had fourth-degree thermal burns caused by flames, with exposure of tendons, bones, and joints, and poor functional prognosis. One third of patients had multiple injuries. Burns affected an average of 36% of the hand surface, and mean coverage was 92.8 cm^2^. One patient died. The 8 others were seen at 30-month follow-up: the skin quality of the flap was found to be good in 55% of the cases, the score on the Vancouver Scar Scale was 2.4, the Kapandji score was 4.5, and total active motion was 37% of that of a normal hand. Hand function was limited in only 2 cases, 8 patients were able to drive, and 3 patients had gone back to work. **Conclusion:** The pocket flap-graft allows preservation of hand function following severe burns, when local or free flaps are impossible to perform. Debulking of the flap at the time of elevation limits the need for secondary procedures.

Primary involvement of the bones and joints, or exposure of partly destroyed tendons, constitutes a rare but serious clinical form of deep thermal injuries to the hand. The estimated frequency is 5% of all hand burns treated in burns centers.^1^ These serious injuries correspond to the fourth-degree burns of Dupuytren's classification.^2^ Because of the thinness of the skin covering, and the injury mechanism, the dorsum of the hand is often injured in adults. In these injuries, coverage by a simple skin autograft, or by an artificial dermis (such as Integra)^3^ followed by a thin skin autograft, is indicated only for very moderate exposures, generally using Kirschner wire fixation.^4^

In extensive exposure, these structures will be subject to drying, infection, and necrosis, which imposes the risk of amputation, unless flap coverage is performed.^5^ A skin or fasciocutaneous flap is the ideal coverage.^6^ In some patients, when a free or locoregional flap cannot be done, an abdominal wall pocket is an alternative that can save the fingers or the hand.^7^ The abdominal wall pocket is an old distant skin flap technique in which a subcutaneous pocket is surgically created for the injured hand, with a view to restoring skin coverage of the dorsum or palm.^8^ To reduce the unsightly and nonfunctional nature of this type of flap, numerous debulking procedures are needed and the use of various techniques has been proposed.^9^ In 1965, Colson and Janvier proposed immediate and total debulking of the distant flaps, and gave them the name flap-grafts.^10^ These flap-grafts were generally utilized for the treatment of burn sequelae and the chosen donor site was on the contralateral arm.^11^,^12^ A variant of this technique, the abdominal flap-graft,^13^ which we name “pocket flap-graft,” is still used in the Percy Burns Center, at the acute phase of certain deep burns of the dorsum and fingers, the main donor site being the abdomen or the homolateral side.

The aim of this study was to determine the value of this technique and whether it is worth pursuing in the future. We focused on the indications and their alternatives, and the short- and long-term results.

## MATERIALS AND METHODS

Our patients were urgent or secondary referrals treated by the pocket flap-graft technique, who were examined using methods of burn wound assessment standardized in recent years.

### Patients

We retrospectively analyzed the medical records of all patients of the Burns Center of the Hôpital d'Instruction des Armées de Percy with thermal injury to the dorsum of the hand who were treated by means of a pocket flap-graft between September 1999 and October 2005. All these patients were asked to attend a medical examination between March and April 2006.

### Methods

We analyzed the patients at 3 stages: hospitalization in the burns center at the acute phase, secondary phase, and recent follow-up examination.

#### Acute phase

Age at the time of injury, sex, principal history, type of burn and its etiology, area burnt, topography of burned areas, and associated injuries were specified for each patient. We considered the burns to be life-threatening when the Abbreviated Burn Severity Index of Tobiasen et al was above 8 and/or the Unit Burn Standard was above 100.^14^

For all hand burns, we noted the injured side, the dominant side, management before pocket flap-graft surgery, injuries indicating surgery (reduced to the number of exposed joints), and the procedures associated with pocket flap-graft management during the acute phase and relating not only to the treated hand (amputations or dislocations, arthrodesis, skin flaps and grafts on the palm or dorsum of the hand).

For each pocket-flap, we noted the location of the coverage on the treated hand, the time (in days) between the date of injury and the date of surgery, the donor site (and whether it was utilized at the same time as the donor site for a thin skin autograft), time to when pedicle was severed, number of stages used to sever the pedicle, method of closure of the donor site, percentage flap survival, and any flap complications.

#### Secondary phase

For each patient we noted the total time spent in the hospital/rehabilitation center, whether the patient was still in the center, the number of sessions of thermal therapy, and the surgical procedures done at this phase.

#### Recent follow-up examination

The information collected comprised time since the burn injury, the type of orthosis, how long compression gloves were worn, interval between the injury and return to work, independence in activities of daily living, driving, satisfaction or dissatisfaction concerning the hand, and discomfort at donor site.

We clinically examined both the treated hand and the other hand, for comparative purposes (except if both hands were treated). The quality of skin coverage was assessed using the following:

The Vancouver Scar Scale (VSS) modified by Baryza and Baryza.^15^ The VSS score comprises 4 variables: vascularity, height, pliability, and pigmentation. Each variable has 4 to 6 possible scores. The scale ranges from 0 to 14, in which 0 corresponds to normal skin.
A simple scale for measurement of the plastic quality of the flap, defined as
  - Excellent (thin, pliable, aesthetic)
  - Good (thin, pliable, but color change)
  - Fair (thick, inadequate hair cover, good mobility, satisfactory dorsal skin reserve)
  - Coarse (very thick, unsightly, but not adherent)
  - Adherent (skin cannot be moved over underlying tissue without dorsal retraction or cutaneous instability)
  - Insufficient (dorsal retraction and/or cutaneous instability)

Any contractures of the web spaces or dorsum were noted. Range of motion was assessed:

**Thumb:** Range of motion for thumb was assessed using Kapandji's score.^16^ When a finger had been amputated, the stump was scored.
**Fingers:** Range of motion for fingers was assessed using the Total Active Motion (TAM) index of the American Society for Surgery of the Hand, proposed in 1976. This index corresponds to the percentage of the sum of the active interphalangeal flexion, minus any extension deficits, compared with the reference TAM, which is the uninjured side (260°, which is the sum of the following range of motion: metacarpophalangeal 80° + proximal interphalangeal 110° + distal interphalangeal 70°). The scale was scored as follows: 100%, excellent; between 90% and 99%, good; between 74% and 89%, fair; below 74%, poor.

The mobility of the extensor digitorum communis was assessed using the British Medical Research Council 0-to-5 scale. The sensitivity over the whole area of the flap was scored as

- superficial tactile fine (cotton),
- deep positional and painful (needle),
- thermal (hot and cold), and
- conventional discrimination (prick-touch).

It was summarized using the British Medical Research Council 5-item classification (S0, no sensitivity; S1, recovery of deep painful sensitivity; S2, recovery of superficial painful and incomplete tactile sensitivity, with hyperesthesia and/or dysesthesia; S3, recovery of complete tactile sensitivity, without dysesthesia, and rough useful discrimination; S3+, recovery of useful discriminatory sensitivity; S4, normal sensitivity).

Hand function was analyzed by means of 5 consecutive exercises: prehension of 3 small objects (pen, bottle top, opening pack of sterile compresses held by other hand) and of 1 large object (full, 1-L bottle of physiological saline) and writing name in lowercase letters (or the first 19 letters if the name is longer). The test was stopped after 60 seconds. The results were classified in 3 groups:

- **Limited:** At least one task impossible to perform within the time allowed.
- **Useful:** All prehension tests completed, but some took longer than 15 seconds; writing done within 60 seconds.
- **Quasi-normal:** All prehension tests done in under 15 seconds, and writing completed in less than 15 seconds (if dominant hand used), or less than 30 seconds in case the nondominant hand was used.

Functional sequelae were considered minor or major, using the classification proposed by Baux.^17^ The minor sequelae were those that resulted in aesthetic impairment, or minor problems likely to improve spontaneously. The major sequelae included at least one functional defect.

### Surgical technique

**Figure 1:**
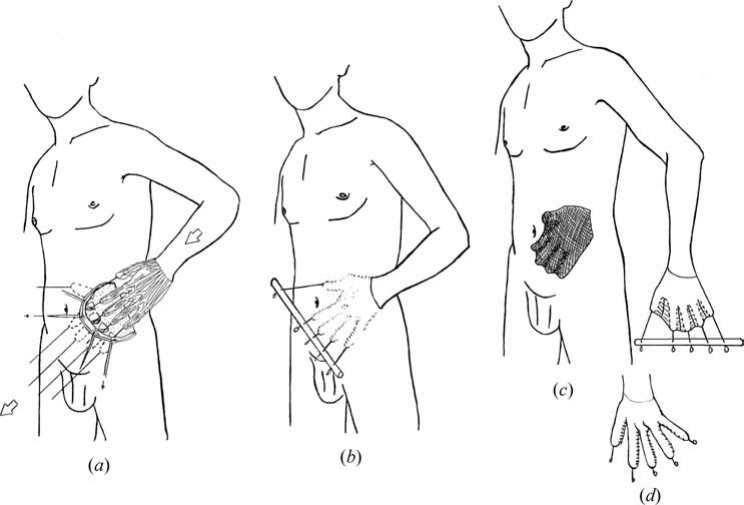
Surgical technique. (a) After excision of necrotic tissue and splinting of the extended fingers with Kirschner wire, the hand was placed in the subcutaneous pocket. (b) The hand was immobilized, and the fingers were separated with an external fixation. (c) After 3 weeks, the hand was cut free from the abdominal wall. The donor site was covered with an autograft. (d) The interdigital webs were separated at the same time, or 2 weeks later, and the Kirschner wires were withdrawn 10 days later.

The surgical technique (Fig 1) was similar to that described by Colson and Janvier.^10^ After excision of necrotic tissue, the fingers were splinted from distal to proximal interphalangeal in extension. When the fingers had to be covered, Kirschner wire fixation stopped at the first phalanx and an external minifixation system was used to maximize finger separation. When the zone to cover was the dorsum, but not the fingers, Kirschner wire fixation continued to the head of the metacarpophalangeal to immobilize it in the “intrinsic-plus” position, fist clenched. The preferred donor site was the abdomen or the homolateral side. When these sites were unavailable, the thigh was utilized. Donor skin area corresponded to the hand surface to be covered (with fingers splayed, when they had to be covered). The burnt hand is put on the zone that will serve as donor site and the best arm position was checked. The patient's hand was used as a model to draw the area to be covered and to make the incision marks. The peeling was done with scissors virtually in contact with the dermis, on skin stretched by Gillis crochet hooks, as in a necklift-facelift. The hand and/or fingers were placed in the subcutaneous pocket, which comprised separate tunnels for each finger, where necessary. The flap was fixed at its ends with a few sutures, and drained by a multitube drain or corrugated silicone drainage sheet (delbet). To ensure flap adherence, the hand was firmly held in position by elbow support using bands. The pedicle was severed from the third week, in 3 steps. Interdigit webs were sectioned along the bisecting line, up to the web space arc. Excess web tissue was removed, except for the dermis, which was sutured to the lateral faces of the fingers.

## RESULTS

Over the last 6 years, we have treated 9 patients (6 men, 3 women; mean age 29.5 years) by the pocket flap-graft technique, among the 1046 patients hospitalized in our department (about half presented with acute second- and third-degree burns to the hands). In 2 cases, the two hands were operated on simultaneously, that is, a total of 11 hands. One female patient died 30 days after suffering the burn injury, that is, 24 days after surgery. Eight patients were followed up (9 hands treated surgically). The last patient of the series was still in the rehabilitation center at the time of the follow-up consultation.

### Acute phase

In all cases, thermal injury was caused by flames, except for one case of burn by contact with hot metal (an iron) (Table 1). On average, 36% of the hand surface was burnt, of which 28% was third-degree. Associated injuries were frequent: 66% inhalation, 33% multiple injuries. Eight of the 9 cases (88%) were life-threatening (Abbreviated Burn Severity Index score > 8).

**Figure 2:**
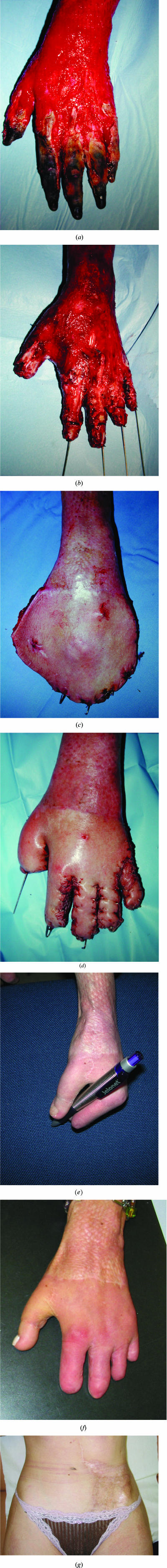
Clinical case 1. (a) Burn to dorsal face of 5 fingers including metacarpophalangeal. (b) Amputation trans second phalanx and proximal interphalangeal arthrodesis after debridement. (c) Appearance before release of fingers. (d) Appearance of flap after separation of fingers. (e and f) Plastic and functional results after toe-to-thumb transfer. (g) Appearance of donor site.

The forearm and arm were burnt on the homolateral side in 9 cases (82%) and also on the contralateral side in 7 cases (63%). The dominant hand was burnt in 7 cases, and in 10 cases (90%) circular deep injuries justified escharotomy. In 2 cases, crush injury was associated with the burn (cases 1 and 4) (Fig 2).

**Figure 3:**
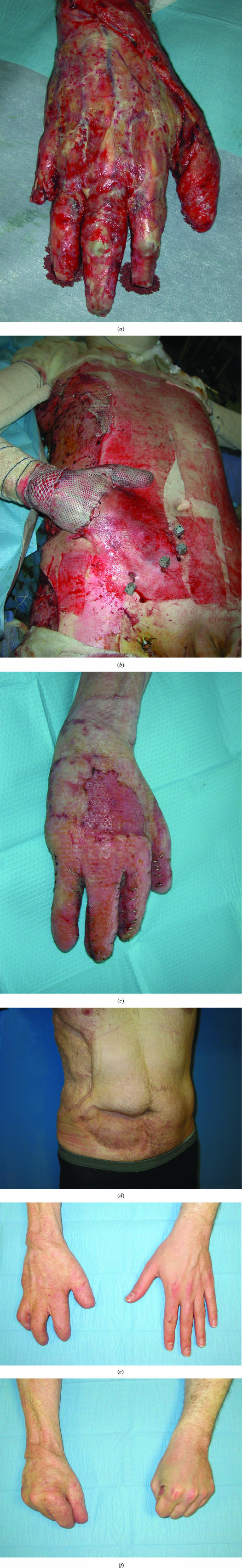
Clinical case 2. (a) Deep burn to whole hand requiring multidigit amputations, appearance after debridement. (b) Defatted pocket-flap on abdomen. The zone utilized for the flap also served at the same time as donor site for thin skin autograft. (c) Appearance after separation of fingers. (d) Appearance of donor site. (e and f) Comparative functional results.

**Figure 4:**
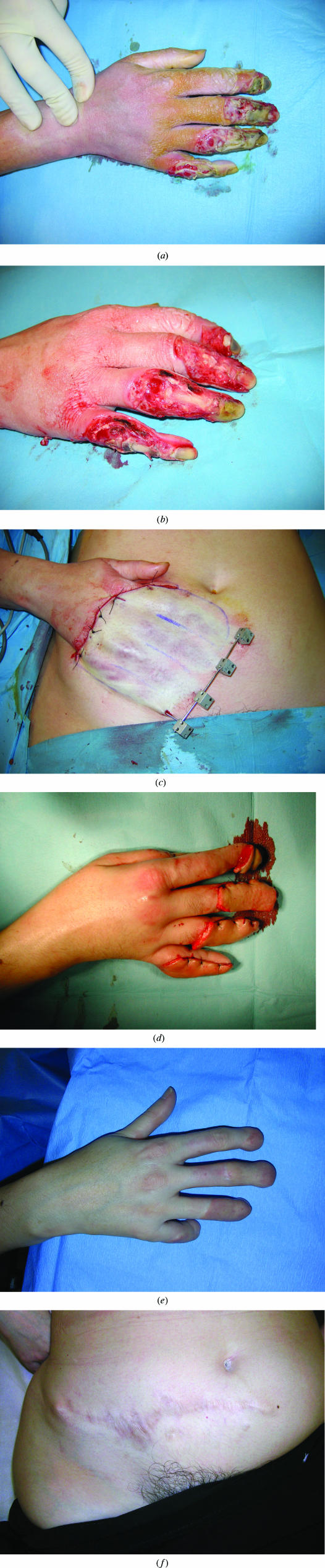
Clinical case 6. (a) Deep burn to distal part of 4 fingers of increasing severity toward cubital fingers, following prolonged contact with a hot iron during an epileptic attack. (b) Appearance after debridement. Note extent of joint destruction. (c) Defatted pocket-flap on abdomen. (d) Appearance after separation of fingers. (e) Appearance of hand at 6 months, without alteration. (f) Appearance of donor site.

**Figure 5:**
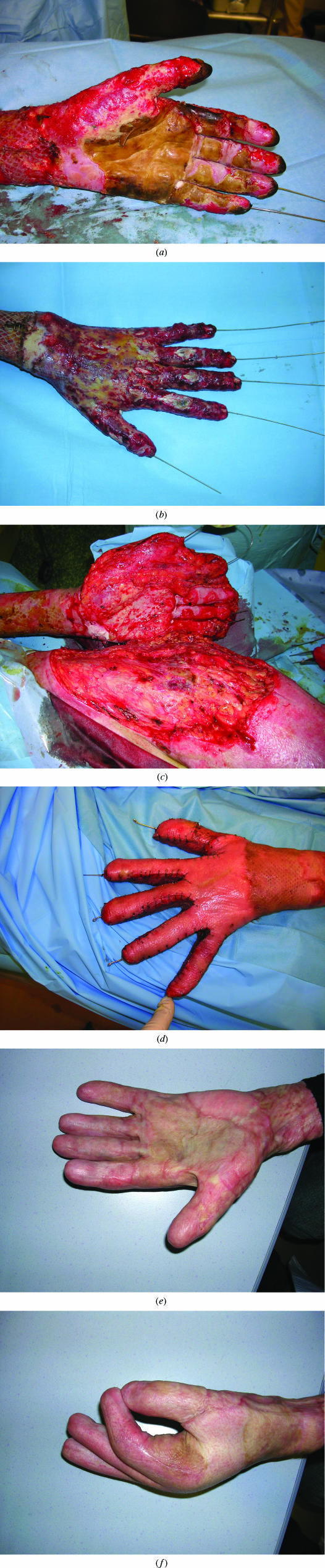
Clinical case 9. (a) Deep burn of the palmar side of the hand. (b) Deep burn of the dorsal side of the hand on day +34 after a failure of Integra. Appearance after excision of necrotic tissue and splinting of the extended fingers with Kirschner wire. (c) The picture shows the hand cut free from the abdominal wall after 7 weeks immobilized in a subcutaneous pocket of the thigh. (d) Appearance after separation of fingers. (e) Appearance of hand at 5 months, without alteration (extension on palmar side). (f) Appearance of hand at 5 months (flexion on radial side).

In 55% of the cases, excision was performed between day 3 and day 5, followed by coverage by artificial dermis (containing bovine collagen) in most cases, or by thin skin autograft, which proved to be a failure. In 3 cases, multiple distal amputations were performed before conducting flap surgery. In 5 other cases, the nail bed of each finger treated was amputated. The nail beds were preserved in a single hand (case 7). On average, 7 exposed joints required flap coverage. In 36% of cases, a posterior interosseous flap was used to complement coverage of the thumb (Fig 3). The fingers were covered in 10 of 11 cases (Fig 4), the dorsum in 4, the thumb in 3, and the wrist in 1 case. The mean area covered was 92.8 cm^2^ (range: 45--160 cm^2^). In all cases, the donor site was chosen on the homolateral side: the abdomen in 6 cases, the side in 3 cases, and the root of the thigh in the last case (Fig 5). The donor site was closed using a thin skin graft in all but one case. The pedicle was severed on average on day 31, and the mean number of surgical interventions, pocket flap-graft included, was 3, that is, on average the pedicle was severed in 2 stages.

All flaps were viable (including 8 cases of 100% survival), even though 3 complications arose. The palm of the hand and/or the lateral faces of the burnt fingers were also covered by a skin autograft in 8 of 9 cases (89%).

### Secondary phase

The 8 patients were examined on average 30 months (range: 5--78 months) after the injury (Table 2). The mean length of stay in the rehabilitation center was 5.1 months, excluding the patient who was still at the center at the time of the follow-up examination (Fig 5). Spa therapy was done 1.3 times (range: 0--1.7) a year on average. The 6 patients who were no longer wearing compression gloves at the time of the examination had worn them for a mean period of 13.3 months (range: 5--24 months).

Surgical interventions during the sequelae (more than a year later) were performed in 5 of the 7 patients (70%) for whom follow-up was sufficient. These interventions (per finger or per region treated) were release of palm metacarpodigital contracture (6 cases), tenolysis (4), debulking of a finger (4), correction of palm web space contracture (3), interphalangeal arthrolysis (2), Atasoy flaps (2) (Fig 4e), interphalangeal arthrodesis (1), toe-to-thumb transfer (1) (Figs 2e and 2f), and treatment of septic arthritis (1).

### Recent follow-up examination

#### Integuments

The plastic quality of the flap was considered excellent or good in 5 of the 9 hands examined (ie, 55%) and fair or coarse in 4 cases (44%) (Table 2). No zone covered by the flap was scored as adherent or insufficient. The VSS score for the skin flap was 2.4 over 14 on average, which is a very good result. No patient complained of pruritus or pain of the skin flap, whereas most complained of these at other sites. In 3 cases, unsightly hair prompted the patient to undertake regular hair removal.

There were 3 cases of web space contracture (graded C1--C4), 2 cases of digit-palm contracture affecting the little finger, 2 cases of digit-palm contracture affecting all the fingers, and one boutonnière deformity.

#### Mobility

The mean Kapandji score was 4.5 (range: 2--10). The TAM score for all fingers was 37% of a normal hand, which corresponds to poor. Clinically, all fingers treated by flap surgery were more or less stiff, at least on extension, and often also on flexion. Most digital motion was provided by the metacarpophalangeal. The proximal interphalangeal and distal interphalangeal joints were more frequently stiff.

The mobility of the extensor digitorum communis was always scored 5, except in case 4, where the score was 0 (which explains the use of a dynamic orthosis by a patient who did not want, for the time being, palliative surgery such as tendon transfer).

#### Sensitivity

In all cases, including the most recent (case 9), the dorsum or the fingers covered by a flap-graft recovered superficial painful and incomplete tactile sensitivity, with slightly useful discrimination, most often at the flap periphery, but sometimes centrally. This sensitivity was always lower than on the opposite side in cases where a single hand was treated, and corresponded to stage S3 of the British Medical Research Council scale at the flap periphery and stage S2 for the whole of the central part. In most cases, thermal sensitivity was absent, except in places where there was slight discrimination.

No difference in sensitivity was noted between the zones treated by posterior interosseous flap (thumb) and pocket flap-graft (fingers) in the 2 cases (2 and 3) where the 2 methods were used simultaneously.

#### Rehabilitation

All patients were independent in all activities of daily living, except in case 9 where the patient needed help putting on compression gloves and positioning facial conformers. Hand function was classified as “quasi-normal” in 2 cases, “useful” in 5 cases, and “limited” in 2 cases. The functional sequelae, according to the Baux classification, were major in all cases.

All patients with a driver's license resumed driving on leaving the rehabilitation center. However, in one case (4), resumption of driving was accompanied by the need to wear a dynamic orthosis, and by the fitting of a ball-grip to the driving wheel. Another patient passed his driving test 1 year after the accident (case 2).

Of the 8 patients followed up, 5 were working before their injury, and of these 2 had resumed work between 7 and 18 months after the acute phase. One patient is completing university training (case 2). One female patient resumed her studies 6 months after the injury and got her first job 4 years after the accident. The 2 patients who had a work accident have not returned to work.

All the patients were pleased that they had undergone salvage surgery, and all reported that any discomfort at the flap donor site was minor.

## DISCUSSION

Our findings show that the pocket flap-graft is a useful method. Above all, it is a surgical alternative that preserves anatomical integrity and some function of the severely burnt hand, when conventional surgical methods are limited and/or difficult because of the general condition of the hand and the extent of the injuries.

The alternative to flap coverage is amputation, which some authors^4^,^18^ advocate when there is tendon destruction, opening of joints, or a destroyed periosteum. Amputation is performed either straight away, when there is vascular and nervous injury, or secondarily after failure of Kirschner wire fixation and skin autograft. These authors reserve flaps for patients who are perfectly stable in intensive care and who have exposed, but intact, tendons and joints.

In our patients, emergency use of flaps was quite rare, and, in general, management was secondary, the indication usually following a failed attempt at coverage by autografts or artificial dermis, in first-line treatment, possibly with Kirschner wire fixation of the fingers. The decision to use flap coverage was taken only if there was no healing in the days after treatment. Conversely, if the tendons were clearly exposed, flap coverage was chosen immediately, as is generally the case in burn centers.^1^,^13^,^19^ The choice of flap depends on the extent of the burn, the availability of local or remote donor skin, and the patient's general condition.^5^

We encountered 2 distinct situations: (1) deep, extensive (90 cm^2^ on average, often affecting the proximal interphalangeal), over 20% third-degree burns, and hence a life-threatening situation, and (2) isolated, deep, multidigit burn. In all cases we used pocket flap-grafts.

*With extensive third-degree burns* (the most frequent situation in our series), the injury was life-threatening and functional concerns were secondary.^20^ This defined treatment procedures and their order. The decision to use excision or flap coverage was taken with care and by assessing its impact on prognosis. The patient's state (shock, infection, instability), the treatments initiated (vasopressins), and the local conditions of the recipient site (local inflammation) contraindicated certain extensive and difficult surgical procedures, such as free flaps. Shen et al used free flaps in acute-phase burns when the average area affected by third-degree burns was 3.2%, and all the patients were perfectly stable.^21^ Also, deep burns to the forearm and to the arm of the burnt hand prevent use of regional, pedicled flaps taken from the forearm.^6^

This very serious situation, which was by far the most frequent in our patients, limited the choice of flaps: groin flap essentially, and possibly tensor fascia lata or intercostal flaps.^22^

The groin flap is the most common distant pedicled flap.^23^ Its vascular reliability is great because of its vascular pedicle being from the superficial circumflex artery, and the length of its pedicle enables some rehabilitation of the hand. Its main drawback is its thickness, and debulking in one or more steps is always necessary. It can, nonetheless, be debulked straight away, and without risk, on its most lateral portion, because from about 5 cm outside the anterosuperior iliac spine the flap is no longer axial but behaves like a random flap, which lives on the dermal and subdermal vascular plexus.^24^

The tensor fascia lata flap with a temporary pedicle has a fasciocutaneous area 3 to 4 times larger than that of the tensor fascia lata muscle. Apart from its thickness and sequelae at the donor site, it has the disadvantage of being taken from the lateral face of the thigh, which is one of the main donor sites for skin autografts in severe burns.^22^

Pedicled intercostal cutaneous perforator flaps, with an oblique or vertical shoulder, have been successfully used for reconstruction of hand scar contractures after burns.^25^

The crane flap procedure^9^ is the last alternative. An abdominal wall pocket is applied to the hand, left in place for 3 weeks, shaved off, leaving a thin layer of subcutaneous tissue, and returning the bulk of the flap to the donor site. After 5 days, the wound granulates sufficiently to receive a skin graft.

*With isolated, deep multidigit burns* (Fig 4, case 6), the problem is very different. Such burns have no general repercussions, and their seriousness is functional. All surgical possibilities, even the most complex, are unrestricted. However, given the quantity of tissue loss to be made up, and its very distal location, the choice of locoregional pedicled flaps is limited, since such a range of action is offered only by radial antebrachial flaps and anterior interosseous flaps.^6^ The homodigital subcutaneous flap for cover of dorsal finger defects is also not indicated, since tissue loss extends beyond the proximal three quarters of the second phalanx.^26^

Another possibility is the free flap. Various donor sites are reported in the literature, and sometimes a cutaneotendinous flap is taken to reconstitute injured tendons: fascia temporalis, flap contralateral radial antebrachial, anterolateral thigh, posterior or lateral brachial, groin, great omentum.^21^,^27^ Of these, the free dorsalis pedis cutaneotendinous flap gives the best functional results when the extensor tendons are destroyed, but at the cost of nonnegligible morbidity at the donor zone.^28^ Vascularized temporoparietal fascia require additional coverage by skin graft, and the morbidity of the donor site is negligible.^29^

### Donor site

We essentially used the abdominal region as the donor site for the pocket flap-graft. The defatted flap is not directly dependent for its circulation on the vessels of the hypodermis, and so can be freely chosen and outlined.^10^

All donor sites described for distant flaps (cross-arm, homolateral or contralateral abdomen, groin, epigastric, subclavicular, inguinal) can be chosen.^10^ Colson considered the cross-arm flap to be the best choice.^12^ We, like other authors,^4^,^13^ consider that the site best for management of severe burns is the homolateral abdomen, or somewhere close, inasmuch as there is often a healthy zone near the abdomen that can be used, unlike the opposite arm, which is frequently burnt. However, the drawback of abdominal skin is its thickness, which decreases moderately with time, and its hair in the case of men.

### Defatting

We defatted our pocket flap-grafts straight away, because the dorsal skin of the hand and of the fingers is normally fine and has no subcutaneous fat.^30^ To optimize finger mobility, the flaps utilized must be as close as possible to normal anatomy, that is, be as fine as possible, with no fat tissue.^10^

The risk of losing the flap through excessive defatting, under the urgent conditions in which they were done, meant that we did not continue defatting beyond the dissection plane of the scissors in contact with the dermis. Colson, on the other hand, defatted in order to obtain the equivalent of a total skin graft (or Wolfe-Krause graft), but did this essentially in surgery of the sequelae.^10^ Our defatting level resulted in a finer primary coverage than with a pedicled groin flap, and also fewer distant debulkings being done.^9^,^22^

Debulking of the pedicled groin flap, as with any single-pedicle flap-graft, is limited to a length of approximately 8 to 10 cm at its most distal portion. Conversely, the pocket flap-graft is bipedicled, and so can be defatted to a length and width of 15 cm to 20 cm.^12^ When one complete hand must be covered, only the pocket flap-graft is defatted over its whole surface.

The pocket flap-graft is a distant flap, and as such has the same advantages and drawbacks.^13^ All distant flaps have the main disadvantage of requiring long immobilization (at least 3 weeks). Besides patient discomfort and nursing difficulties, there is a risk of stiffening of the joints of the hand, elbow, and shoulder. This risk was not investigated in the present study. The other disadvantages are the need for several surgical procedures, and parasitic vascularization at the recipient site after the pedicle is severed, unlike free or locoregional pedicled flaps.

The foreseeable advantages of distant flaps are their relative simplicity of use, compared with flaps with vascularized skin and tendon. The lack of a homogeneous study population and common assessment criteria in the relevant literature prevented us from comparing our overall results with findings for other types of flaps, but some comparisons are nevertheless possible in terms of technique and outcome.

### Reliability

Our pocket flap-grafts' reliability was good and comparable to that of flap-grafts.^12^,^13^ The risk of failure was greatest with free flaps and smaller with groin flaps. Shen et al noted complete necrosis in 13% of cases,^21^ and Guiga et al reported partial and distal necrosis in 2.5% of cases.^22^

### Quality of skin coverage

Long-term skin coverage was good, as confirmed by the VSS score and the absence of dorsal retraction. Burns to the dorsum frequently result in hypertrophic scarring and dorsal retraction,^2^ due to a lack of skin. The skin on the dorsum must have an extra 2 to 3 cm after coverage of a burn, and the loss of this extra skin prevents finger flexion. The use of a flap seems to avoid this type of outcome, confirming the good VSS score and the absence of dorsal retraction, in comparison with retraction of the palm or web space. Despite the length of use of compression gloves, and the number of sessions of thermal therapy, the VSS scores were quite uniform and good.

### Sensitivity

The recovery of sensitivity is less important for the dorsum than the palm but should not be overlooked. The pocket flap-graft allowed recovery of protective sensitivity. Our results agree overall with literature findings on the recovery of sensitivity of distant flaps.^31^,^32^ Note that the sensitivity of distant flaps, as the groin flap, is equivalent.

### Functional sequelae

Patients were examined at different times after injury (range: 5 months to 5 years), which may explain observed differences, notably in the possibility of functional recovery.

The TAM index for the fingers and the Baux classification of functional sequelae^16^ are widely used, but we feel they are not suited to this type of injury, as almost all patients were put in the same category, although some had seemingly better recovery than others.

At follow-up, we utilized our own hand function assessment, which can therefore only be compared in a limited way. We were unable to use the Jebsen-Taylor Hand Function Test,^33^ because although it has the advantage of being used extensively in literature reports, it is time-consuming and requires many equipment items.

It is also difficult to analyze our surgical technique in isolation, insofar as it is combined with other methods that contribute to its success (physiotherapy, equipment, continuous wearing of compression gloves, thermal therapy, plastic surgery of sequelae).^17^ Likewise, it is hard to assess the results of this surgical technique without also taking into account the impact of the associated injuries, which were very frequent in our patients. Sherif and Sato noted that hand burns plus burns over more than 20% of the body surface (as here) were also accompanied by a significant increase in sequelae. They adduced factors as the depth of the initial injury (often affecting several anatomical regions), but also rehabilitation difficulties and multiple surgical interventions.^34^

We used pocket flap-grafts on hands that had suffered more or less severe destruction of the tendons and/or joints. Nuchtern has shown that fourth-degree burns of the hand have significantly more functional sequelae than third-degree burns (in Dupuytren's classification),^4^ as confirmed by Sheridan et al, who found that hand burn victims with fourth-degree burns were significantly more likely to be dependent in activities of daily living.^1^

The medium- and long-term functional results we obtained were nonetheless relatively satisfactory, when compared with failure to preserve anatomical integrity, that is, amputation. After multidigit amputation, functional loss is greater with shorter fingers. Likewise, the surgical possibilities, in terms of secondary repair (replacement of fingers by metacarpals, toe-to-thumb transfer), diminish.^35^

It is unanimously agreed that this rescue surgery reduces the sequelae of this type of burn, as confirmed by Nuchtern et al who reported that fourth-degree burns treated by flaps (irrespective of technique) healed significantly quicker, and had significantly less marked functional sequelae, than those treated by the alternative method of delayed closure with immobilization by Kirschner wire fixation and skin grafting.^4^

Finally, we did not examine in depth the psychological impact of this rescue surgery, but the initial results have been satisfying and we encourage continued use of the pocket flap-graft technique.

## CONCLUSION

The pocket flap-graft is well suited to the coverage of extensive burns to the dorsum of the hand and fingers, with exposure of tendons or joints, because of the free choice of donor site (within the range of motion of the hand), complete coverage of the dorsum, simplicity, which is an advantage in the early management of severe burns, reliability, fineness, and protection of the palm in nutrient medium to promote healing when the palm too is burnt. We therefore consider this method to be an alternative of choice in preserving hand function, when other methods of coverage are not possible. This is why, despite its nursing requirements, this technique should continue to be used at the Percy Burns Center. Our findings now require confirmation in a comparative prospective study.

## Figures and Tables

**Table 1:** Characteristics of the population at inclusion and flap results in the acute phase*

	Case								
	1	2	3	4	5	6	7	8	9
Age, y	21	30	26	33	43	40	30	25	18
Sex	F	M	M	M	F	F	M	M	M
Etiology	Flame (RA)	Flame (RA)	Flame (autolysis)	Flame (fire, WA)	Flame (fire)	Iron	Flame (fire, WA)	Flame (RA)	Flame (RA)
Burnt area,%	20	40	55	25	30	2	43	50	60
Background	…	Asthma	Schizophrenia, substance abuse	…	Chronic obstructive pulmonary disorder, alcohol abuse smoking, depression	Epilepsy	Depression	None	None
Associated injuries	Multiple injuries: face, spine, thorax, abdomen, limbs	Inhalation	Severe inhalation	Inhalations, injury to thorax, abdomen, spine, crush syndrome	Severe inhalation	…	…	Inhalation, multiple injuries to thorax, abdomen, brain	Inhalation
Burn topography	Face, neck, upper limbs, thighs	Head, neck, back, upper limb and right lower limb	Head, neck, hands and wrists, lower limbs, buttocks	Back, right upper limb, legs	Head, thorax, abdomen, back, upper limbs, thighs	Hand and right wrist	Head, neck, back, upper limbs thighs	Face, neck, upper limbs, lower limbs, buttocks	Head, neck, thorax, back, upper limbs
Hand	Left†	Right†	Left	Right†	Right	Left†	Right†	Right†	Right†	Left	Left
Level of amputations	Trans P1 thumb, trans P2 and arthrodesis IP 4 fingers	DIP from D2 to D4 and MP D5	IP thumb, MP D2, DIP of D3 and D5	…	…	…	…	…	…	…	…
Number of joints exposed	4	3	4	5	9	9	9	1	9	9	6
Flaps and arthrodesis	PIP arthrodesis 4 fingers	PIF‡ on thumb	PIF on thumb	…	PIF‡ on thumb	PIF‡ on thumb	…	…	…	…	…
Coverage location	Dorsum, 5 fingers	4 fingers, MP included	Dorsum, 4 fingers	Wrist and dorsum	4 fingers	4 fingers	4 fingers	D1, D2, and D3, MP included	4 fingers, 4 fingers and IP thumb	Dorsum, 5 fingers
Area covered, cm^2^	81	80	90	130	72	80	45	68	115	100	160
Time to flap surgery	D+27 (transfer)	D+23 (failure of Integra)	D+5	D+37 (failure of Integra)	D+6	D+9	D+44 (failure of graft)	D+36 (failure of Integra)	D+34 (failure of Integra)
Donor site	Abdomen	Abdomen§	Abdomen	Side	Side	Abdomen	Abdomen	Abdomen	Thigh
Pedicle severed	W4, W5, and W6	W3 and W4	W3 and W4	W4		W4 and W5	W3	W4 and W5	W7
Donor site closure	Graft	Graft	Graft	Graft		Direct suture	Graft	Graft	Graft	Graft
Flap complications	…	…	Partial necrosis	…		…	Infection	…	…	Hematoma
Flap survival	100%	100%	80%	100%		100%	100%	100%	100%
Thin skin autografts	Lateral and palmar faces of fingers	Thumb, dorsum, lateral and palmar faces fingers	Dorsum, palm, palmar faces of fingers	Dorsal face of thumb		Cubital faces of D4 and D5, hypothenar eminence	Lateral faces of fingers	Lateral and palmar faces 4 fingers	Lateral face 4 fingers, palm, palmar face D1, D2, and D5

*… indicates none; RA, road accident, WA, work accident; PIF, posterior interosseous flap; MP, metacarpophalangeal; IP, interphalangeal; PIP, proximal interphalangeal; DIP, distal interphalangeal; D2, second finger; P1, first phalanx; W, week; and D, day.

^†^Dominant side.

^‡^Fasciocutaneous flap taken from burnt forearm.

^§^Flap-graft also utilized as donor site for thin autograft.

**Table 2:** Rehabilitation, treatment of sequelae, follow-up, and outcome*

	Case								
	1	2	3	4	5	6	7	8	9
Follow-up, mo	78	61	50	29	27	20	13	5
Length of stay in rehabilitation center, mo	6	4	10	5	External	2	9	4 (ongoing)
Number of courses of spa therapy	9	8	6	4	0	3	1	0
Wearing of compression gloves, mo	24	24	6	5	7	14	10 (ongoing)	4 (ongoing)
Hand	Left	Right	Left	Right	Right	Right	Right	Left	Left
Secondary surgery	Left toe-to-thumb transfer, release of palm contractures C1 and C2, and digit-palm, fingers	Arthrolysis PIP D3 and D4, tenolysis D3 and D4, debulking D3 and D4	Treatment of septic arthritis of MP, release of contracture of palm MP	…	Release palm contracture MP D5, PIP arthrodesis and DIP D5, Atasoy flap on D4 and D5	…	…	Debulking and tenolysis D2 and D3, correction contracture C1	…
Plastic quality of flap	Good	Good	Poor	Excellent	Excellent	Good	Gross	Fair	Fair
Vancouver Scar Scale score	2	2	3	1	2	3	3	3	3
Retractions and contractures	Digit-palmar web space of D5	Digit-palm, contracture of web spaces D1–D4	Claw fingers (severe DP retraction)	…	Digit-palm web space D5	Boutonniere deformity D3, nail bed dystrophy	Contracture of web spaces C1–C4	Contracture of web spaces C2–C4	…
Kapandji score	5#	5#	3#	3#	10	7	3	3	
TAM index	Poor	Poor	Poor	Poor	Good	Fair	Poor	Poor	Poor
TAM index, middle finger	Poor	Poor	Poor	Poor	Good	Fair	Poor	Poor	Poor
TAM index, ring finger	Poor	Poor	Poor	Poor	Fair	Fair	Poor	Poor	Poor
TAM index, little finger	Poor	…	Poor	Poor	Poor	Fair	Poor	Poor	Poor
Function	Hand useful	Hand useful	Limited movements	Hand useful with dynamic orthosis	Hand quasi- normal	Hand quasi- normal	Hand useful	Hand useful	Limited movements
Driving	Yes	Yes	No	Yes (ball-grip)	Yes	Yes	Yes	No
Returned to work (after how long)	Secretary, job adapted (4 y) Studies (6 mo)	LTL student (8 mo)	HA	LTSL	Secretary, same job (7 mo)	Sick leave	Computer scientist, same job (18 mo)	No

*LTL indicates long term leave; HA, handicap allowance, LTSL, long-term sick leave; TAM, total active motion; ^†^, dominant side; #, Kapandji score determined for finger stumps; MP, metacarpophalangeal; IP, interphalangeal; PIP, proximal interphalangeal; DIP, distal interphalangeal; D1, thumb; D2, second finger; D3, middle finger; D4, ring finger; D5, little finger; DP, digit-palm; C1, first web space; C2, second web space; C4, fourth web space; y, year; and mo, month.
